# Probing the physicochemical characteristics of carrot sauce during storage

**DOI:** 10.1371/journal.pone.0273857

**Published:** 2022-11-16

**Authors:** Muhammad Sameem Javed, Adnan Amjad, Faiz-ul-Hassan Shah, Zulfiqar Ahmad, Aneela Hameed, Muhammad Junaid Anwar, Ammar Ahmad Khan, Muhammad Amir, Muhammad Jawad, Muhammad Abrar

**Affiliations:** 1 Institute of Food Science and Technology, Bahauddin Zakariya University, Multan, Punjab, Pakistan; 2 Department of Food Science and Technology, Faculty of Agriculture and Environment, The Islamia University of Bahawalpur, Bahawalpur, Punjab, Pakistan; 3 University Institute of Diet and Nutritional Science, The University of Lahore, Lahore, Punjab, Pakistan; 4 Post Harvest Research Centre, Ayub Agricultural Research Institute Faisalabad, Faisalabad, Pakistan; Bangabandhu Sheikh Mujibur Rahman Agricultural University, BANGLADESH

## Abstract

Globally, the prevalence of vit-A deficiency disorders i.e., xerophthalmia and nyctalopia is increasing especially in teenagers due to lifestyle shifts and undernutrition. This research was designed to develop carrot-supplemented tomato sauce to overcome vit-A deficiency and its related disorders. The carrot sauce was formulated with the addition of 50, 60, and 70% carrot pulp in tomato paste. The prepared sauce samples were tested for physical and biochemical changes in beta carotene (BC), lycopene, viscosity, pH, total soluble solids, titratable acidity, total plate count, and sensory parameters for 12 weeks. A non-significant effect of storage on BC, lycopene, and total soluble solids was observed. The total plate count, acidity, pH, and viscosity were influenced significantly. Sauce containing 60% of the carrot paste showed good sensory characteristics and 42.39 μg/g BC for the whole period of storage. It is concluded that carrot sauce can be used as tomato ketchup replacers to boost the overall quality of life by fighting against vit-A deficiency disorders.

## 1. Introduction

Chronic diseases due to micronutrient deficiencies are the major public health concerns [1]. With the increasing malnutrition, food safety issues, and pandemics health budgets are increasing in developing countries. According to Dawn Newspaper published on 28^th^ August 2021 [2], only in Pakistan, has the health budget increased 49.6% (14.5 in 2020–21 to 21.7 billion Rupee). World Health Organization (WHO) reported that 53% of school-going children are suffering from the vit-A deficiency that leads to night blindness, distortions in color differentiation and anaemia. Vit-A deficiency is more prevalent in underdeveloped countries [3]. In Pakistan, 43% of the total population is facing vit-A deficiency with compromised physical activity among growing children. Recommended dietary intake of vit-A for children (6 to 12 years) is 250–350 μg as basal and 400–600 μg as safe, set by FAO/WHO [4]. Vit-A deficiency is the main cause of public health risk and needs attention to combat through supplementation and manufacturing of novel food products containing high bio-available vit-A contents [5].

In the era of processed foods across the cosmopolitan markets, the enrichment of the most popular products (i.e., sauces and ketchup, etc.) is a wise strategy to feed all age groups suffering from a vit-A deficiency. Different studies suggested that the average consumption of sauces varies between 17–25 g/day [6]. Tomato sauce is the most popular commodity formulated from tomato paste, vinegar, sweeteners, spices, flavoring agents, and some types of hydrocolloids including carboxymethyl cellulose [7]. Among the vegetables, carrots are rich in carotenoids that are the natural precursor of vit-A. Carrots contain 3.2 to 170 mg kg^-1^ carotenoids, 24.97 μg/g BC and 21 to 775 mg kg^-1^ vit-C [8]. Different carrot-based products i.e., smoothies, milk, chips, and yoghurt in which BC contents were 173.19 ± 1.02 μg/g, and carrot pomace supplemented tomato sauce were developed to reduce the risk of vit-A deficiency disorders [9]. The concentrations of BC do not change significantly after cooking as proved by the comparison of raw carrots with boiled and puree of carrots [10]. The digestion of carotenoids starts in the stomach and are absorbed via the intestinal brush border and move into the bloodstream as a chylomicron [11]. In Pakistan, Punjab province contributes the largest share of carrot production about 326.63 million tons. Punjab province contributes a major part (approximately 67%) in the economy of agriculture products where carrot production adds 2.8%, which is 68% higher compared to other provinces [12]. The most popular varieties i.e., red and purple colored contain higher amounts of vit-A in the form of α- and β-carotene. Bio-fortification of carrots improves the carotene content above 70% in various products [13]. The research was hypothesized that the supplementation of carrots to the sauce will enhance its nutritional profile and have the potential to reduce vit-A deficiency disorders.

In the country, carrot is mostly used to make fresh juice and carrot-based sweet dessert pudding (Gajar ka halwa) which are seasonal. Sauces are used round the year which can reduce the effect of seasonality and are a better option to supplement with more nutritious ingredients. The current study aimed to develop the carrots supplemented tomato sauce and to assess the effect of storage on its nutritional composition and shelf-life.

## 2. Material and methods

To optimize the sensory attributes of sauce, carrots and tomatoes were organically raised in the town of district Vehari, Pakistan. These vegetables were then harvested at horticultural maturity levels early in the morning. Vegetables were washed properly and transported in ice buckets to the food processing laboratory of the Institute of Food Science and Nutrition, Bahauddin Zakariya University Multan, Pakistan. In the laboratory, carrots were sorted, trimmed, and washed to remove inedible parts.

### 2.1. Preparation of carrot sauce

The carrot supplemented sauce was prepared by following the [14] with slight modifications. After coring the carrots were chopped and blended by laboratory-scale blender LB20ES at 9500 rpm to form a fine pulp. Tomatoes were washed and pulped by the same blender at variable speed. The sauce was prepared by using different concentrations of carrot and tomato pulp (Table 1) in a non-stick pan adding the measured quantities of onion, garlic, ginger, coriander, red chillies, sugar, black pepper, sodium benzoate, salt, vinegar, and corn flour. After concentration, the pulp was homogenized using stainless steel homogenizer (colloid mill), sieved and heated to final consistency. The product was aseptically filled in glass jars and stored at room temperature for further analysis. All samples were analyzed for below mentioned physicochemical analysis at regular intervals of 15 days for the period of 3 months.

**Table 1:** Replacement of tomato pulp by carrot paste in various concentrations.

Treatment	Carrot %	Tomato %
T_0_	0	100
T_1_	50	50
T_2_	60	40
T_3_	70	30

### 2.2. Physicochemical analysis

Proximate tests along with physicochemical and sensory analysis of the product samples were done by following the methods given in AOAC [15].

### 2.3. Determination of β-carotene

Almost 1g of representative sample was taken in a test tube and 5 mL acetone was added into it and kept for 15 min followed by intermittent shaking at high speed for 10 min. The test sample was then subjected to centrifugation for 10 min at 1370 relative centrifugal force. Two successive supernatants were separated in a test tube by adding 5 mL acetone in a test tube for each. The supernatant obtained was mixed with already obtained supernatant and filtered with Whatman filter paper No. 42. The absorbance of the sample was measured at 454 nm by using a spectrometer [16].

### 2.4. Determination of lycopene

Carrot sauce was weighed (5–10 g) in the beaker and acetone ether was used to extract the sample repeatedly until colorless. The sample was then transferred into a separating funnel, added 10–15 mL of petroleum ether and thoroughly mixed. Carotenoid pigments found in the acetone were transferred to the petroleum ether by diluting with distilled water and 5% sodium sulphate-containing acetone. The solution was then centrifuged at 3000 rpm using M-800 LT– 161210 centrifuge machine. The supernatant was moved into the other funnel to achieve colorless sample and discard the acetone step. The petroleum ether was added to the 50 mL volumetric flask and diluted to label with petroleum ether and readings were observed at 503 nm by using spectrophotometer [15]. Lycopene present in the sample was calculated by given formula.


$$Lycopene\;(mg/g)=\frac{Sample\;absorbance\times OD\;of\;sample\times vol.made\;up\times 100}{\;1\times Wt.of\;sample\times 1000}$$

OD = Optical Density = 3.1206

### 2.5. Viscosity determination

The viscosity of carrot sauce was determined by using LVT viscometer purchased from Brookfield engineering laboratories USA. The sample was weighed (200g) in a 250 mL beaker and attached the suitable spindle to the viscometer. The spindle was fully dipped into the sample at its described mark. Readings were taken from the dial when the needle was in the stationary phase after 5–6 rotations. To calculate the factor by using rpm and spindle number it was multiplied with readings [17]. The viscosity of carrot sauce was determined by the following formula:

Dial reading × Factor = Viscosity in cP

Factor = rpm × spindle number

CP = centipoise (Unit of viscosity)

### 2.6. Determination of plate count

For inoculum preparation, 1 mL from each sample was added in test tubes containing 4 mL of distilled water. Nutrient agar along with Petri-plates was sterilized in an autoclave (Hirayama HVA-85). The prepared media was then poured into plates in a vertical type of laminar airflow cabinet (CJ-1D). After streaking, the plates were incubated at 37°C in Memmert IF750 incubator for 48 hours and then bacterial colonies were counted by digital colonometer (FJ-2/FJ-3) in log cfu/mL by following the method described in AOAC [15].

### 2.7. Determination of total soluble solids (˚Brix)

Total soluble solids of carrot sauce were measured by using a portable refractometer (Manufactured by MRC) at 20°C [15]. Before measuring, the prism was rinsed with distilled water and cleaned using tissue paper. The drop of sauce was placed on the prism and readings were taken.

### 2.8. Determination of pH

pH readings of carrot sauce were taken by using a digital pH meter (Model Iino-Lab720 Germany). The pH meter was calibrated by buffer solution of 4, 7 and 9. The sauce was taken in a 25 mL beaker and successive readings were directly noted from the screen by inserting the electrode in the sample as per the given protocols of AOAC [15].

### 2.9. Total titratable acidity

The titratable acidity of carrot sauce was determined by the simple titration method described in AOAC [15]. The samples were titrated by 0.1N sodium hydroxide against ascorbic acid and titratable acidity was calculated by using given equation

$$Total\;Acid\;\%=\frac{\frac{1}{10}\times Eq.weight\;of\;acid\times Normality\;of\;NaOH\times Titer}{10}$$

### 2.10. Sensory evaluation

Sensory evaluation of carrot sauce was conducted by a trained panel, students and staff members. The samples were served in the sensory analysis lab of the Institute of Food Science and Nutrition, Bahauddin Zakariya University under white light to each evaluator along with potato chips. The parameters like color, texture, taste, aroma, mouthfeel and overall acceptability were recorded by using a 9-point hedonic scale (extremely like = 9 and extremely dislike = 1) by following the method provided by Everitt [18].

### 2.11. Statistical analysis

Statistical analysis of data was carried out to check the level of significance by using statistical software statistix 8.1. Liner model was used to conduct analysis of variance of the data by applying a two-factor factorial design taking three replicates and two variables i.e., carrot: tomato concentrations (4 levels) and storage days (7 levels) under CRD at 0.05% confidence interval [19].

## 3. Results and discussion

### 3.1. Storage stability of β-carotene

The storage study of 12 weeks was conducted to evaluate the stability of β-carotene (BC) in carrot sauce. Data analysis depicted that BC contents were slightly influenced during the whole period of storage as shown in Table 2. BC contents were 10 times higher in a sample containing 70% carrot paste than control at the initial level of storage. The decrease in BC concentration was ~ 24.6% at the end of the 12-week storage study. The effect of treatments and storage was highly significant while a non-significant effect was observed for the interaction between treatments and storage. BC contents in T_1_ containing 50% carrot paste were 43.02 ± 2.60 at 0 days of storage and observed 38.56 ± 1.34 at12^th^ week. A significant reduction in BC contents was linked with the effect of light, oxidative stress, temperature fluctuations and packaging [20]. The BC contents in the sauce sample containing 60% carrot paste were 49.97 ± 2.80 before storage which reduced to 42.39 ± 2.17 (15.16% reduction). A similar decreasing trend was observed in T_3_ i.e., 46.39 ± 2.83 at the end of storage. Carotenoids are located inner to the cell membrane and are highly sensitive to oxidation when stored under ordinary conditions. The porosity of the cell membrane due to an increase in temperature may leach carotenoids [21]. BC stability studied by Mehrad *et al*., [22] was also negatively correlated with storage conditions and encapsulation matrix.

**Table 2:** Storage study of 12 weeks to determine the β-carotene stability in treatments.

Treatments	Storage weeks						
	0	2	4	6	8	10	12
**T**_**0**_	5.75^k^±0.49	5.60^k^±0.51	5.25^k^±0.18	5.04^k^±0.15	4.98^k^±0.14	4.77^k^±0.18	4.33^k^±0.35
**T**_**1**_	43.02^ej^±2.60	41.96^gj^±1.70	41.27^gj^±1.51	40.50^hj^±1.12	40.04 ^ij^±0.93	39.81^IJ^±0.78	38.56^j^±1.34
**T**_**2**_	49.97^ad^±2.80	49.14^ae^±3.10	48.20^af^±2.93	47.37^bg^±3.34	45.81^ci^±1.90	44.75^di^±0.90	42.39^fj^±2.17
**T**_**3**_	53.85^a^±3.27	52.22^ab^±3.11	51.42^ac^±2.96	50.72^ad^±1.71	49.56^ad^±0.66	49.05^ae^±1.11	46.39^bh^±2.83

### 3.2. Storage stability of lycopene

Lycopene is a bright red coloured compound that on heating isomerizes to give dark red colour. During the study period a non-significant effect of storage observed on lycopene contents while a highly significant difference was found among the treatments (Table 3). Maximum lycopene contents were noted in control and minimum in a sample containing 70% carrot paste. Lycopene contents approximately remained unchanged till the end of the storage period showing a 0.93% decrease. In the sample containing 50% carrot paste, the lycopene contents were 93.09 ± 0.36 μg/g at 0 days of storage and observed 92.12 ± 6.12 μg/g at the end of storage. Lycopene contents in T_2_ of the fresh product were maximum which decreased ~1.71% till the end. Similar trends were observed for the sample containing 70% carrot paste. Smaller changes in the lycopene may be due to isomerization during heat processing and oxidation which convert the trans isomers into cis isomers thus reducing bioactivity. Light, heat, acids and various other factors contribute to the degradation of lycopene. The results were coherent with the findings of Li *et al*., [23]. Martínez-Hernández *et al*., [24] also observed a negative correlation between the days of storage and lycopene contents but not to a significant extent. According to the data reported by Liang *et al*., [25] the minor degradation in lycopene present in tomato pulp can be prevented by forming emulsion using different oils i.e., olive oil.

**Table 3:** Storage study of 12 weeks to determine the lycopene stability in treatments.

Treatments	Storage weeks						
	0	2	4	6	8	10	12
**T**_**0**_	153.26^a^±0.26	152.9^ab^±0.29	152.7^b^±0.23	152.5^bc^±0.18	152.2^cd^±0.21	152.0^cd^±0.18	151.83^d^±0.26
**T**_**1**_	93.09^e^±0.36	93.02^ef^±0.22	92.90^ef^±0.13	92.75^efg^±0.11	92.56^fgh^±0.19	92.31^gh^±0.15	92.12^h^±6.12
**T**_**2**_	87.05^i^±0.26	86.90^ij^±0.13	86.66^ij^±0.28	86.42^jk^±0.32	86.00^kl^±0.18	85.79^l^±0.11	85.56^l^±4.14
**T**_**3**_	79.33^m^±0.51	78.93^mn^±0.50	78.68^no^±0.55	78.50^nop^±0.50	78.27^opq^±0.39	78.18^pq^±0.33	78.00^q^±0.26

### 3.3. Effect of storage on titratable acidity of carrot paste supplemented tomato sauce

Titratable acidity (TA) is a measure of total acids present in a product and is responsible for colour, taste and microbial stability. Storage and treatments had a highly significant effect on the TA. From Table 4, it is evident that the TA of carrot sauce was slightly changed with the storage time. In the control sample, the TA of the sauce was changed significantly with the increase in storage period and was 2.14 ± 0.04 at the 12^th^ week. TA of carrot sauce having 50% carrot paste increased to 10% during three months of storage period and was 1.52 ± 0.02 at the initial level which increased at the end of storage. In treatment having 60% carrot paste the value of TA changed from 1.45 ± 0.02 to 1.71 ± 0.015 during the period of 12 weeks. In T_3_ having 70% carrot paste the value of TA at 0 days was 1.40 ± 0.025 which increased to 1.45 ± 0.02, 1.58 ± 0.02 and 1.68 ± 0.03 at 4^th^, 8^th^ and 12^th^ week. This positive correlation between TA and storage time might be due to the breakdown of polysaccharides, sugars oxidation and degradation of pectic substances. A higher amount of salts in the carrot sauce may be another reason for increasing of TA [26]. A study conducted by Pourfarzad & Derakhshan [27] on hazelnut sauce also showed that TA increased during storage while using guar gum and tragacanth as a thickening agent.

**Table 4:** Storage study of 12 weeks to determine the titratable acidity in treatments.

Treatments	Storage weeks						
	0	2	4	6	8	10	12
**T**_**0**_	1.85^e^±0.03	1.91^d^±0.02	1.94^d^±0.01	2.02^c^±0.04	2.08^b^±0.03	2.12^ab^±0.03	2.14^a^±0.04
**T**_**1**_	1.52^n^±0.02	1.54^mn^±0.01	1.57^klm^±0.02	1.59^ijk^±0.01	1.64^h^±0.02	1.68^g^±0.02	1.69^f^±0.02
**T**_**2**_	1.45°±0.02	1.50^n^±0.01	1.54^mn^±0.01	1.55^lm^±0.01	1.58^jkl^±0.02	1.6300^hi^±0.01	1.7133^fg^±0.015
**T**_**3**_	1.40^p^±0.025	1.44^op^±0.01	1.45°±0.02	1.51^n^±0.02	1.58^jkl^±0.02	1.62^hij^±0.03	1.68^g^±0.03

### 3.4. Changes in pH of carrot paste supplemented tomato sauce

Changes in pH value of carrot sauce during the period of three months are given in the mean Table 5. A decreasing trend in pH of the sauce was observed throughout the storage period. The effect of treatments was significant on the pH of the sauce while the interaction between storage and treatments was non-significant. In the control sample the value of pH at the start of storage was 3.68 ± 0.08 which decreased to 3.55 ± 0.03 at the 4^th^ week of storage and this trend continued till the end with a value of 3.37 ± 0.02. This reduction in pH of the sample having 50% carrot paste was noticed from 3.81 ± 0.02 at the initial level to 3.66 ± 0.08 and 3.48 ± 0.02 at the 6^th^ and 12^th^ week of storage respectively. In T_2_ pH at the initial level of storage was 3.55 ± 0.60 which increased till the 4^th^ week and then gradually decreased to 3.47 ± 0.12 at the end. But in the sample having 70% carrot paste the pH showed a decrease of 7.7% till the end of storage. Prakash *et al*. [28] reported a pH range of 3.25 to 3.65 in acerol tomato ketchup. Moringa root supplemented tomato sauce also indicated similar results i.e., 4.62 ± 0.05 which reduced with the progression of storage [26]. The changes in pH of the carrot sauce may be correlated with microbial invasion and oxidative breakdown of complex compounds thus releasing organic acids. Environmental factors also contribute to biochemical changes during storage of the product.

**Table 5:** Storage study of 12 weeks to determine the pH stability.

Treatments	Storage weeks						
	0	2	4	6	8	10	12
**T**_**0**_	3.68^a-g^±0.08	3.58^a-j^±0.03	3.55^d-j^±0.03	3.47^g-j^±0.06	3.44^hij^±0.04	3.39^ij^±0.02	3.37^j^±0.02
**T**_**1**_	3.81^a^±0.02	3.79^ab^±0.02	3.72^a-e^±0.3	3.66^a-h^±0.08	3.59^a-j^±0.04	3.56^c-j^±0.03	3.48^f-j^±0.02
**T**_**2**_	3.55^d-j^±0.60	3.78^abc^±0.08	3.74^a-e^±0.6	3.67^a-g^±0.3	3.61^a-i^±0.04	3.52^e-j^±0.12	3.47^g-j^±0.12
**T**_**3**_	3.76^a-d^±0.10	3.73^a-f^±0.10	3.66^a-h^±0.1	3.63^a-h^±0.1	3.57^b-j^±0.14	3.54^d-j^±0.14	3.47^g-j^±0.14

### 3.5. Effect of storage on total plate count of carrot supplemented tomato sauce

The correlational study between different treatments and storage time indicated that the highest total plate count (TPC) was observed in treatment having 70% carrot paste at 12^th^ week of storage. From the Table 6 it is evident that the minimum value was found in control at the start of storage which increased at 12^th^ week. A similar increasing trend was seen in T_1_ during the storage showing an elevation of 12.22%. The value of TPC in treatment having 60% carrot paste was noted 2.98 ± 0.1 Log CFU/g at 0 days and showed a highly significant increase during the whole period i.e., 3.40 ± 0.07 Log CFU/g at 12^th^ week. According to the set parameters by WHO/FAO 7 Log CFU/g of TPC is safe for human consumption with exception of *Salmonella*, *E*. *coli* and *coliforms* [29]. The results were in coherence with the previous study of Sucharitha *et al*., [30] who reported an increase in TPC of tomato preserve from 2.27 to 8.15 for one month. Microbial growth may be occurring due to the different thickening agents and the type of storage condition. The presence of carbohydrates may also support microbial growth [31].

**Table 6:** Storage study of 12 weeks to determine the total plate count in treatments.

Treatments	Storage weeks						
	0	2	4	6	8	10	12
**T**_**0**_	1.48^k^±0.22	1.54^jk^±0.25	1.58^jk^±0.23	1.59^jk^±0.19	1.63^jk^±0.19	1.66^jk^±0.19	1.79^j^±0.14
**T**_**1**_	2.37^i^±0.29	2.44^hi^±0.29	2.42^hi^±0.31	2.46^hi^±0.26	2.55^hi^±0.25	2.62^hi^±0.23	2.70^gh^±0.24
**T**_**2**_	2.98^fg^±0.1	2.96^fg^±0.05	3.04^ef^±0.06	3.11^def^±0.07	3.20^c-f^±0.06	3.32^a-e^±0.07	3.40^a-d^±0.07
**T**_**3**_	3.17^c-f^±0.06	3.25^b-f^±0.03	3.28^a-e^±0.06	3.35^a-d^±0.08	3.44^abc^±0.06	3.49^ab^±0.08	3.55^a^±0.06

### 3.6. Variability in total soluble solids (°Brix) of carrot supplemented tomato sauce during storage

During three months, treatments and storage have a highly significant effect on total soluble solids (TSS) as depicted in Table 7. The effect of treatments and the interaction between storage and treatments was highly significant. The lowest value was observed in the control sample at the initial level of storage which increased gradually till the end. In treatments having 50 and 60% carrot paste, the value of TSS was not changed during the whole period. A slight change was observed in T_3_ and values changed from 27.95 ± 1.75 to 29.11 ± 1.87. Environmental factors and acid hydrolysis of polysaccharides are considered major contributors to fluctuations in TSS [32]. The value of TSS for strawberry ketchup ranged from 26.1 to 27. 3 [33] and TSS reported for Brazilian ketchup was in the range of 24.36 to 33.35 [34].

**Table 7:** Storage study of 12 weeks to determine the total soluble solids in treatments.

Treatments	Storage weeks						
	0	2	4	6	8	10	12
**T**_**0**_	24.79^j^±1.23	24.93^j^±1.39	24.89^j^±1.83	25.03^j^±1.28	24.91^j^±2.19	24.95^j^±1.83	25.18^ij^±1.31
**T**_**1**_	25.93^fg^±1.41	26.15^f^±1.82	25.70^gh^±1.03	25.75^gh^±1.71	25.51^hi^±1.48	25.67^gh^±1.29	25.98^fg^±1.52
**T**_**2**_	26.87^de^±2.00	27.03^d^±1.45	26.89^de^±1.90	27.01^d^±1.52	26.72^e^±1.29	27.17^d^±1.62	27.05^de^±1.91
**T**_**3**_	27.95^c^±1.75	28.07^c^±1.69	27.96^c^±2.15	28.25^c^±1.84	27.93^c^±1.45	28.69^b^±1.72	29.11^a^±1.87

### 3.7. Effect of storage on the viscosity of sauce

All the analyzed samples of different treatments showed that the viscosity of carrot sauce decreased during storage. Initially, the control sample was more viscous than other treatments i.e., 2286.0 ± 52.37 which reduced to 1312.3 ± 50.64 at the end of storage (Table 8). Statistical analysis revealed that a significant difference exists between viscosity, storage weeks, and treatments (P < 0.05). A decreasing trend was observed in all treatments i.e., in T_3_ viscosity was 1589.7 ± 86.23 at 0 storage week which gradually reduced to 736.0 ± 5.91 at the 12^th^ week. Xu *et al*. [35] reported that different processing methods and storage affect the viscosity of different tomato-based products. Conversions of pectic polysaccharides present in carrots are contributing to a decrease in viscosity. The results showed a similarity when different thickening agents were used to optimize the moisture retention properties of tomato sauce [36].

**Table 8:** Storage study of 12 weeks to determine the viscosity in treatments.

Treatments	Storage weeks						
	0	2	4	6	8	10	12
**T**_**0**_	2286.0^a^±52.37	2100.7^bc^±138.91	2014.7^cd^±115.94	1925.7^def^±51.05	1721.7^gh^±71.30	1515.7^ij^±3458	1312.3^kl^±50.64
**T**_**1**_	2184.6^ab^±36.75	1984.7^cde^±175.92	1848.3^efg^±95.37	1596.7^hi^±188.21	1512.0^ij^±29.86	1296.7^kl^±63.10	1092.7^mn^±227.06
**T**_**2**_	1789.3^fg^±66.50	1700.7^gh^±58.77	1596.7^hi^±73.56	1285.0^kl^±34.39	1214.7^lm^±46.52	1023.7^n^±161.55	789.0°±147.40
**T**_**3**_	1589.7^hi^±86.23	1511.7^ij^±5095	1377.7^jk^±38.39	1218.7^klm^±65.59	986.0^n^±9.53	736.0°±40.85	736.0°±95.91

### 3.8. Sensory attributes of the sauce

#### 3.8.1. Colour changes during storage of carrot supplemented tomato sauce

**Fig 1:**
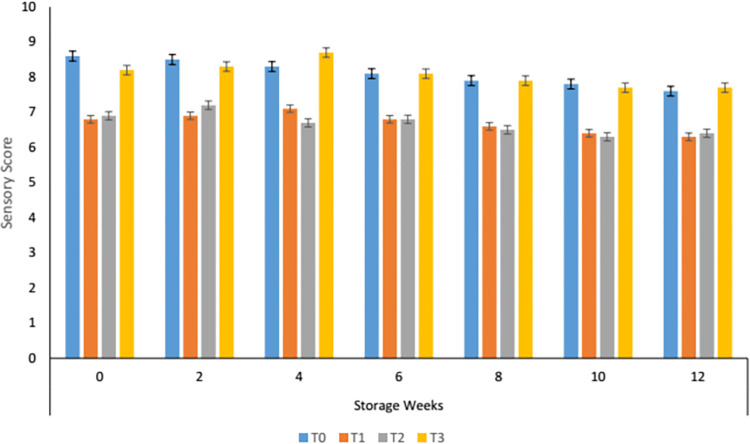
Sensory colour of carrot sauce.

Sensory color recorded by the panelists indicates that the highest score was given to the sample having 70% carrot paste followed by control. Variation in color values among the treatments is prominent while a non-significant effect of storage was noted (Fig 1). The sensory color increased slightly till the second week of storage and then continued decreasing. The declining pattern might be due to the oxidation and isomerization of β-carotene during storage. The reducing trend was observed in all the treatments but stability was noted in T_3_. Li *et al*. [23] linked the color changes with the degradation of lycopene and Maillard reactions. Data reported by Kaur *et al*, [37] stated that different processing and storage conditions affected the color of the tomato hot pot. Consequentially T_3_ was best among the others with more nutritional value and β-carotene contents.

#### 3.8.2. Textural variations in carrot supplemented tomato sauce during storage

**Fig 2:**
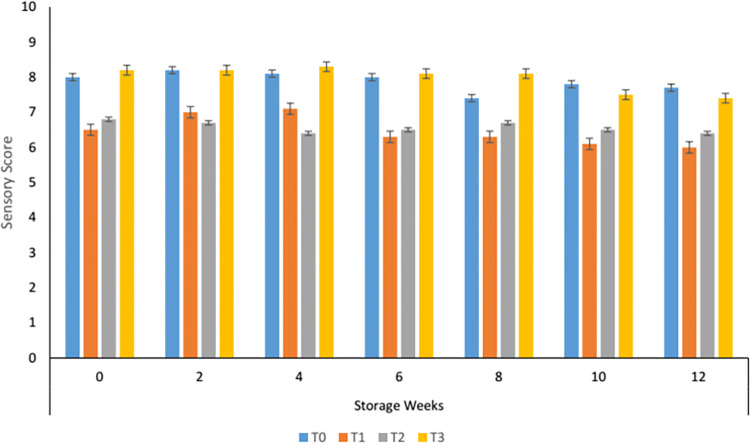
Texture of carrot sauce.

Textural evaluation of the carrot sauce showed that more smoothness was observed in control and T_3_ at the start of storage. In the sample having 50 and 60% carrot paste sensory texture was significantly different from the control and with 70% carrot paste, also a similar was observed with the progression of storage. Textural instability was more in samples containing carrot paste as compared to control (Fig 2). A slight clumping was observed leading to textural changes. According to Szczesniak, [38] textural changes in plant-based products are due to the breakdown of pectic substances and polysaccharides which on disintegration may contribute to clump formation to some extent. Similar results were reported by Kamsina & Anova, [39] when Kandis acid was used in the formulation of tomato sauce.

#### 3.8.3. Effect of storage on the taste of carrot supplemented tomato sauce

**Fig 3:**
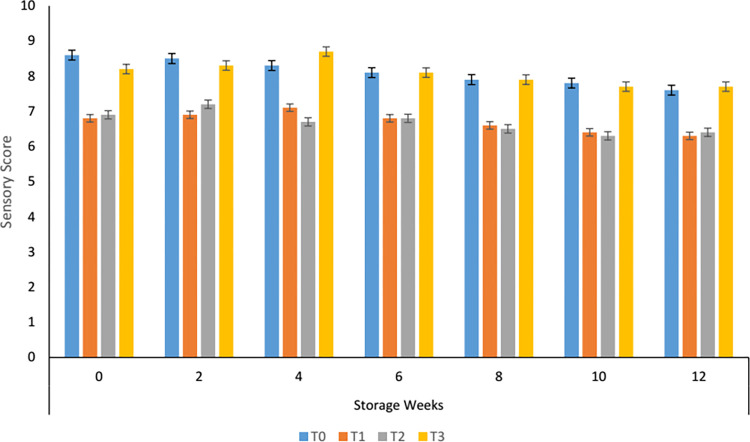
Taste of carrot sauce.

The sensory taste of the carrot sauce was significantly different among the treatments. A comparable taste score was evaluated in the control sample and T_3_ while in T_1_ and T_2_ this score was not accepted by the panelist (Fig 3). With the progression of storage, taste improved in the sample having 70% carrot paste till the 4^th^ week and then starts decreasing gradually but found more satisfactory than the rest of the treatments and control samples. Nkhata & Ayua [40] reported that taste was negatively influenced after one month when stored at 30°C while no adverse effect was found at 6°C. Similar, results were observed for the moringa root supplemented tomato sauce [26].

#### 3.8.4. Effect of storage on mouth feel of carrot supplemented tomato sauce

**Fig 4:**
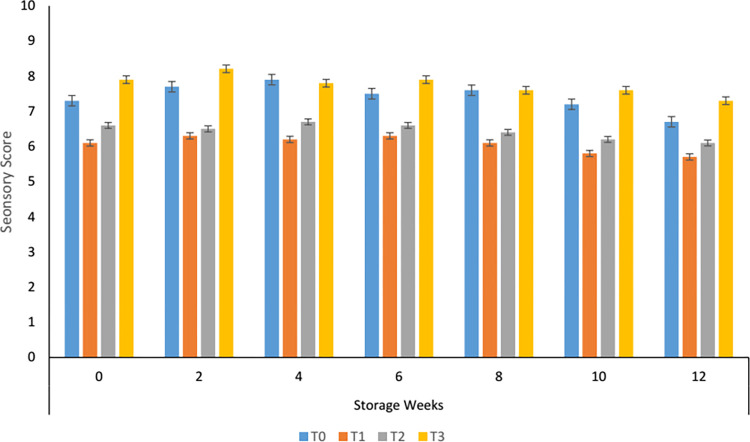
Mouthfeel of carrot sauce.

Panelist scores depicted that the mouth feel of carrot-based sauce increased till the 2^nd^ week and then start decreasing progressively. It is clear from Fig 4. That among the treatments T_3_ was best when compared with control and found satisfactory till the end of storage. The treatments had 50 and 60% of carrot paste were significantly different (P < 0.05) from control and T_3._ Xu *et al*. [41] monitored a significant reduction in mouth feel while studying the post-harvest changes in tomatoes. Landy *et al*. [42] also found that there was no correlation between mouthfeel and storage days but environmental conditions negatively influenced mouth feel.

#### 3.8.5. Effect of storage on the aroma of carrot supplemented tomato sauce

**Fig 5:**
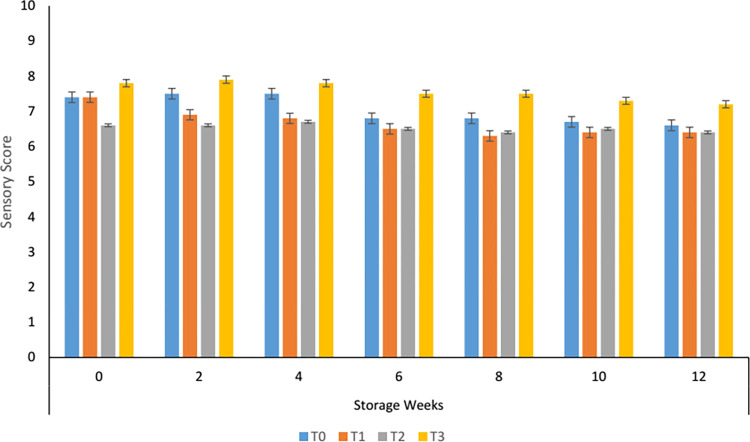
Aroma of carrot sauce.

Aroma is one of the sensory attributes directly perceived by the consumer. During the study period, the effect of different treatments and storage conditions on the sensory attributes of carrot sauce was significant (Fig 5). The highest aroma score was observed in the sample having 70% of carrot paste when compared with control and remained in the acceptable range till the end of the storage period. Throughout the storage T_1_ and T_2_ not yielded the best. The results of the study were in corroboration the findings of Din *et al*., [26].

#### 3.8.6. Overall acceptability of carrot supplemented tomato sauce

**Fig 6:**
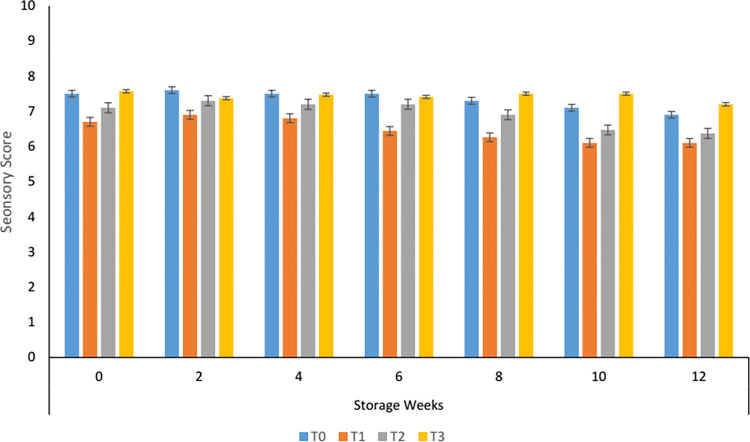
Overall acceptability of carrot sauce.

Data analyzed by the sensory panelist for the overall acceptability of the carrot sauce supported that a highly significant difference was found among the treatments (P < 0.05). The effect of storage was less prominent during the whole period (Fig 6). The maximum acceptable range was observed in T_3_ and then in the control sample. The overall acceptability of samples having 50 and 60% of carrot paste was not satisfactory at the end of storage. Din *et al*., [26] reported a similar while storing moringa root supplemented tomato sauce for 90 days with a value of 7.46. A similar reducing trend in the overall acceptability of tomatoes during post-harvest changes was observed by Landy *et al*. [42]. Conclusively, carrot sauce having 70% carrot paste behaved well during the whole storage period with the good sensory and nutritional profile.

## 4. Conclusion

The study was focused to develop a carrot-based sauce to enhance the β-carotene contents and assess the changes in the composition of the sauce. As sauces are mostly utilized with fried products to enhance flavor and taste. The product was stored for 12 weeks and evaluated at 15 days intervals to check the effect of storage on its nutritional composition, shelf stability, and consumer acceptance. The study proved that sauce containing 60% carrot paste showed better sensory and textural attributes as compared to control. The deteriorative changes in T_2_ were also lesser as compared to other treatments. It was concluded that carrot supplemented tomato sauce can be used as a functional food for the betterment and treatment for the vit-A deficient population.

## Supporting information

S1 Data Raw results carrot sauce. (XLSX)
